# Some of the Mistakes of Surgical Gynecology

**Published:** 1896-06

**Authors:** T. A. Stoddard

**Affiliations:** Pueblo, Colo.; Gynecologist to the Pueblo Hospital


For the Texas Medical Journal.

### SOME OF THE MISTAKES OF SURGICAL GYNECOLOGY.

BY T. A. STODDARD, M. D., PUEBLO, COLO.
Gynecologist to the Pueblo Hospital.

Read at the Fort Worth Meeting of the Texas State Medical Association, April 29, 1896.

WE ARE met here in the interests of suffering humanity, and not, as generally supposed by the laity, for our own personal aggrandizement or the furtherance of our own selfish aims.

The subject I have chosen, is one both broad and deep, and if you will excuse me, I will try to avoid the deep, keeping myse f confined to the broad, so that I may have sufficient latitude to say what I wish, without getting beyond my depth.

You will pardon me if I appear somewhat dogmatic in what I

have to say on this subject, but personal experience and observation in this line of work, has a tendency to either dogmatize or disgust one. We have seen men risk and ruin their reputations, and bring discredit upon our noble profession, we have seen valuable lives lost or rendered miserable or valueless by egotistical attempts at operative interference in unsuitable cases, or by men whose operative skill was in an embryonic state, if it was not entirely absent. Such are the sins of commission. Again we have seen many cases urgently demanding operative interference, which have been temporized with, treated with opiates, and on "the expectant plan." Such are the sins of omission. We see and hear every day of operations which only serve to laud the skill of the operator, without benefitting the patient. Egotism, ignorance and jealousy are the factors which contribute more than all others to these mistakes to which I will call your attention for a few minutes.

1. It is a mistake to promise too much in the way of results. Very many of the cases which demand operative interference are so complicated that only the charlatan would dare promise anything but a possible benefit. Take, for instance, some intra-abdominal pathological condition, and before the abdomen is opened, we can not be positive of the exact condition existing. I have in mind a case to which I was called by a brother practitioner, who informed me that it was a case of acute obstruction of the bowels, due to the patient having swallowed a number of plum stones. The obstruction had been complete for twenty-three days. I opened the abdomen, and found cancer of the sigmoid flexure, involving at least six inches of bowel. I performed colotomy at once, thus saving the patient for a time, but she eventually succumbed to the malignant growth. Here was a case that, according to the family physician, gave no history of any previous trouble, and yet there was incurable disease. I would have made a sad mistake if I had promised a cure. And yet, many good men are making this mistake constantly, to their own personal and professional injury, and to the discredit of the profession at large.

2. It is a mistake to ask a patient to allow you to operate in preference to some one else. This is an evidence of want of self-respect, and even if the results are satisfactory, we are likely to receive contumely. Again the case may be worse than we anticipated, and we are not able to give the results we promised--for the first two mistakes go together—and then I can conceive of no more ridiculous and painful a position than that occupied by the operator. If our patient needs an operation, let us tell her so, and, if need be, urge her to have it done, but don't let us ask her to give us the preference.

3. It is a mistake to do a major operation when a minor one will serve the purpose as well, or better.

Plastic work in the vagina has, of late, been much neglected by the rank and file of the profession, as well as by those who are considered leaders. This is, no doubt, due in a large measure to the desire on the part of operators to have a large list of major operations. Much can be done by judicious plastic work to correct displacements of uterus bladder and urethra, and to overcome many of the painful pelvic symptoms which are erroneously thought to be due to diseased vicera. The modified barbarous Alexander's operation has fortunately, to a great extent, been relegated to "the things that were," except by a few immortals, who have gone wild over the "vaginal route." Hysterrhaphy will doubtless soon occupy a still more obscure position, after it has done much to damage surgical work in this field. Hysterectomy is done when operations of much less magnitude would serve the purpose as well so far as the patient is concerned. The abdomen is being opened for simple displacements, which, under proper treatment, would require no operation. We seem to have become madly insane in this matter of major operations, and the sooner a halt is called the better for humanity.

4. It is a mistake to think that any particular method of operating is the only correct one in all cases. Take for example the different operations for repair of a lacerated perineum. We have Werth's, Hegar's and Hildebrandt's, Goodell's, Stude's and Voos', Simon's, Bichoff's and Tait's, and a host of others too numerous to mention. Now, the operator who will blindly follow any of these methods without a conception of the eternal fitness of things, has no right to be allowed to attempt such work. In all the field of surgery there is no department which demands greater originality than this. There are scarcely two cases alike, and, therefore, few cases can be successfully operated upon by following any set rule of stitch and suture. Have we not all been more or less disgusted with some of our teachers of and writers in surgery. One will say that the Tait operation is the proper one, and will explain fully how it is done, carcely mention any other method, and thus leave the student under the impression that he has received the "*sumum bonum*" of the method of repair of all cases of lacerated perineum. Some of these so-called methods are absolutely pernicious and misleading, and show only a desire on the part of the originator to get his name before the public, and to narrow the opening into the vagina, both of which are accomplished with great *eclat*.

The choice of methods for removal of the uterus or its appendages must be left to the operator in each particular case, and the dictum of the advocates of this, that or the other method must be ignored entirely, except in an elective way, and the manner of operating, as well as the line of after treatment of the stump of the patient, must be decided by the operator in relation to the existing pathologic condition, and the peculiarities of the case.

Every case demands careful study and intelligent use of anatomical and surgical knowledge and principles, without regard to Prof. A.'s or Dr. B.'s method. The man who has no mechanical ability, but must do everything by rule of "thumb," has no more right to attempt operations of this character than the quack who has no knowledge of anatomy.

5. It is a mistake to sacrifice blood and tissue for the sake of rapidity.

Prolonged anæsthesia is certainly to be deprecated and shock to be guarded against, but to my mind shock is not produced by loss of blood, or by reason of lengthy operations. Shock, I firmly believe, is a purely reflex condition, or rather a result of reflex action, and may be produced by injury to the Solar plexus or not. If the anæsthetic is carefully administered until reflexes are completely quiescent and no work done while these are active and care exercised in handling the abdominal vicera, I believe that this bugbear of the surgeon will cause him less concern in the future than in the past. Get the patient in the best possible condition, and then take time to do the operation *well*. If an operation is not done as well as it can be done, it is not done well enough, and nothing will excuse a man in doing an operation half way right, not even a want of time or fear of shock. Be sure and secure all bleeding vessels, for there is no doubt that many deaths attributed to shock have really been due to loss of blood. Some years ago, among my first operations, was one where I removed a large ovarian cyst which had many adhesions. I secured all bleeding points and left the patient in charge of the House Surgeon, whom I asked to notify me if any unfavorable symptoms arose. About two hours later I was telephoned that shock was profound. I went in all haste, opened up the wound and found severe hemorrhage, due to slipping of a ligature. I secured the vessel which was in the omentum, again closed up the abdomen, and the patient made a slow but uneventful recovery. This taught me a lesson which I never forgot.

6. It is a mistake for any physician to attempt major operations unless he has thoroughly prepared himself, by work of a similar character on the dead. At the present time it seems to be a fad for every one, general practitioners and all, to attempt any or all the work which has come to be considered special.

This is brought about by two causes:—(a) many place themselves before the public as specialists, without the necessary qualifications, and are therefore no more fitted for the work than the general practitioner. This is not as it should be, and there is as much need of protecting the public against such men as against the veriest quack, who goes from town to town. Some begin the practice of a specialty as soon as they have graduated, without ever having done any general practice whatever. The men who do this are dishonest with their patrons and discourteous to their medical brethren. (b) The second cause is that men who have prepared themselves for special work still do general practice, and thus insult their *confreres* in general practice by tacitly saying, "I am as good a general prctitioner as you, and I can do special work better." Now, while there may be some show of truth in this, still it is insulting, and I contend that the man who places himself before the public as a specialist in certain lines, should give up general practice for his patients' sake and for his brother practitioners' sake. It is hardly reasonable to expect the general practitioner to send his special cases to the specialist who does general practice as well. For this cause operations are attempted by incompetent men, or operations are delayed until too late, and thus an injury is done to all. October last I was called in consultation by a general practitioner to see a lady who was supposed to have had a miscarriage a few days before. The doctor had been in attendance four or five days, and the day on which I was called he had curetted the uterus for supposed retained placenta. Shortly after the curettment the patient began sinking, and eight hours after I was called and found the patient in extremis and every evidence of collapse from hemorrhage. There was dullness over the lower part of the abdomen. I diagnosed ruptured tubal pregnancy and refused to operate, first because the patient was too far gone, and second because the family physician did not agree with me in the diagnosis. The patient lived only a few hours, and I was fortunate enough to secure an autopsy, which proved the correctness of my diagnosis. The placenta was located toward the outer end of the tube, and here the tube had ruptured, allowing the blood to escape into the abdominal cavity. The fœtus was apparently well formed and about the fourth month. The history of this case should have led any one to suspect the true state of affairs, and had I been called in before the curettage this life might have been saved. There seems to exist in the mind of the general practitioner an antipathy for all specialists. There is no doubt that if the general practitioner were endowed with the skill of a Michael Angelo, he would paint the gynecologist knee deep in the almond-shaped ovulating glands of the human female, a scalpel in one hand and a ligature carrier in the other, surrounded by blear-eyed assistants, whose desire for gore and gland is only exceeded in intensity by that of their principal. Possessing this spirit I have seen men go to operations whose nerve consisted of corn-juice and ignorance, and dared to lift over the bared abdomen that instrument so potent for good, but equally potent for harm, and I have trembled for the profession. We can not get by reading alone what is necessary to render us capable of doing with success and in the best possible manner any operation, however simple.

7. It is a mistake to attribute to electricity the virtues many claim for it. The weight of opinion the world over is that the use of electricity in the treatment of fibromata and myomata has done little good and much harm, and that too when used by men who thoroughly understand its therapeutic application; but when we consider the many who use this agency without any knowledge of its use or abuse, then and only then, will it appear before us in its proper light. True, there are some men of ability who claim for this agency some excellent if not wonderful results, but against these are arrayed a long list of the most eminent men who ever graced the profession of medicine, men of honor, men of science, and men of research, who, after having given this a fair trial, have discarded it as useless, if not positively harmful. When a fibroid has reached such a size as to cause symptoms, the only proper course to pursue is to remove it by surgical means, and not subject the patient to the dangers of sepsis and delay. They tell us, certainly, of numerous cases of cure by electricity, but so have we heard of cancers cured by Christian Science, and "falling sickness" by faith cure.

It rests with us as specialists to set the example of being careful and conservative. Many misconstrue the word conservative. It is no. conservative to perform a hysterectomy when a plastic vaginal operation will do better; neither is it conservatism to treat a pus tube or an abscess of the ovary by local application alone. It is not conservative to do Alexander's operation, when a curettment, in nine cases out of ten, will serve the same purpose; neither is it conservatism to apply for months the electric current to a bleeding fibroid. It is not conservative to curette the uterus for every case of painful menstruation, neither is it conservative to give a tonic and advise change of air for a case of deep-seated purulent endo-metritis.

Let us be conservative, but let us also be honest with our patients, honest with ourselves, and honest with those who learn from us or are taught by us. Let the teachers in our colleges be chosen because of their fitness, and not because of any political *pull.* Let them be men, sober, painstaking and broad-minded, slow to follow fads, and deeply grounded in the humanitarian principles of the great profession of medicine. and then we may expect from the rising generation of physicians more men of mentality, and fewer of those who grasp at every fad which is foisted upon the profession, and who act as barnacles to impede our progress.

